# The Potential of a Brown Microalga Cultivated in High Salt Medium for the Production of High-Value Compounds

**DOI:** 10.1155/2017/4018562

**Published:** 2017-05-22

**Authors:** Saoussan Boukhris, Khaled Athmouni, Ibtissem Hamza-Mnif, Rayda Siala-Elleuch, Habib Ayadi, Moncef Nasri, Alya Sellami-Kamoun

**Affiliations:** ^1^Laboratory of Enzyme Engineering and Microbiology, University of Sfax, National Engineering School of Sfax, BP 1173, 3000 Sfax, Tunisia; ^2^Laboratory of Biodiversity and Aquatic Ecosystems, Ecology and Planktonology, Department of Life Sciences, Sfax University, Soukra Km 3.5, BP 1171, 3000 Sfax, Tunisia

## Abstract

*Amphora* sp. was isolated from the Sfax Solar Saltern and cultivated under hypersaline conditions. It contains moderate rates of proteins, lipids, sugars, and minerals and a prominent content of bioactive compounds: polyphenols, chlorophyll a, carotenoids, and fatty acids. The analysis of fatty acids with GC/MS showed that the C16 series accounted for about 75% of* Amphora* sp. lipids. Saturated fatty acids whose palmitic acid was the most important (27.41%) represented 41.31%.* Amphora* sp. was found to be rich in monounsaturated fatty acids with dominance of palmitoleic acid. It also contains a significant percentage of polyunsaturated fatty acids with a high amount of eicosapentaenoic acid (2.36%). Among the various solvents used, ethanol at 80% extracted the highest amounts of phenols and flavonoids that were 38.27 mg gallic acid equivalent and 17.69 mg catechin equivalent g^−1^ of dried extract, respectively. Using various* in vitro* assays including DPPH and ABTS radicals methods, reducing power assay, and *β*-carotene bleaching assay, the 80% ethanolic extract showed high antioxidant activity. A strong antibacterial activity was checked against Gram-positive bacteria (*Staphylococcus aureus* and* Micrococcus luteus*) and Gram-negative bacteria (*Klebsiella pneumoniae* and* Salmonella enterica*). These results are in favor of* Amphora* sp. valorization in aquaculture and food and pharmaceutical industries.

## 1. Introduction

Microalgae are considered as an important source of bioactive compounds with a wide range of applications and commercial use. They produce proteins, lipids, vitamins, pigments, and other molecules exploited for health, for food and feed additives, and for energy production. Due to their wide diversity and high storage capacity for lipids, particularly eicosapentaenoic acid (EPA) and amino acids, microalgae have pharmaceutical and cosmetic applications [1, 2]. Furthermore, the protective effects of natural antioxidants, against chemically induced oxidative damage, have been paid attention nowadays, especially when the generation of free radicals is involved. Benthic diatoms have become good sources of natural antioxidants, as revealed by a number of studies [3–6]. Moreover, microalgae represent a large and underexplored resource of antimicrobial compounds [7, 8]. Potentially, microalgal extracts are already being tested as additives for food and feed formulations, in an attempt to replace the currently used synthetic antimicrobial compounds, including subtherapeutical doses of antibiotics employed as a prophylactic measure in animal breeding [9]. Microalgae are ubiquitously distributed throughout the biosphere, where they have been adapted to survival under a large spectrum of environmental stresses, such as heat, cold, anaerobiosis, salinity, photooxidation, osmotic pressure, and exposure to ultraviolet radiation [10]. Hence, they may grow essentially under most of the environmental conditions available, ranging from freshwater to extreme salinity. They possess the extra advantage of substantial metabolic plasticity, dependent on their physiological state. Likewise, their secondary metabolism can easily be triggered by most forms of externally applied stress [7].

Among the large variety of microalgae, Cyanophyceae, Chlorophyceae, Bacillariophyceae (diatoms), and Chrysophyceae are the most studied for biodiesel production [11]. It is estimated that diatoms produce about 25% of the global primary biomass [12]. In the Sfax Solar Saltern, Bacillariophyceae dominated with Dinophyceae in the least salty ponds but they are rarely abundant in hypersaline environments [13].

Unlike the universal macromolecules of primary metabolism (e.g., monosaccharides, polysaccharides, amino acids, proteins, and lipids), which are commonly present in all organisms, secondary metabolites have a far more limited distribution in nature. They are not necessarily produced under all conditions and can only be found in specific organisms or a group of organisms. The organism can produce these compounds either to protect itself within its own living ecosystem or to play a basic role in its everyday existence. This reveals bioactive molecules in unrelated biological systems [14]. The medicinal and pharmacological actions are often dependent on the presence of bioactive compounds called secondary metabolites [15]. The growth of algae is relatively rapid, and it is possible to control the production of their bioactive compounds such as polyphenols and pigments by modifying culture conditions [10].

In the present work,* Amphora* sp. (Bacillariophyceae) was cultivated in a hypersaline medium and the physicochemical properties, fatty acids profile, bioactive compounds, and some biological activities were determined.

## 2. Materials and Methods

### 2.1. Microalga

*Amphora* sp. is a Bacillariophyceae that was isolated via micromanipulation and serial dilution from a water sample collected in the pond C4-1 of the Sfax Solar Saltern with average salinity of 107 p.s.u. (practical salinity unit). The Sfax Solar Saltern is located in the central eastern cost of Tunisia, at about 34°39′N and 10°42′E. The ponds are shallow (20–70 cm depth) and have various salinities ranging from 40 to 400 p.s.u. [16]. They are connected by pipes and channels along a 12 km section of sea cost.

### 2.2. Culture Conditions

The microalga was cultivated in batch in autoclaved artificial seawater, which was enriched with F/2 nutrient medium, sodium silicate (Na_2_SiO_3_), and trace metals solution [17, 18]. Cultures were conducted for 15 days at the salinity of 100 p.s.u., 25°C, light : dark (L : D) cycle of (16 h : 8 h), and cool white fluorescent light intensity of 60 *µ*moles photons m^−2^ s^−1^. The biomass was separated from the culture media by centrifugation (4500 ×g, 10 min), and the pellet was washed with distilled water and centrifuged again at 4500 ×g for 10 min (the washing was repeated two times). The pellet was freeze-dried and stored at −70°C.

### 2.3. Determination of Dry Matter, Ash, Proteins, Lipids, and Total Sugars

The dry matter and ash content were determined according to the AOAC standard methods [19]. Total nitrogen contents of* Amphora* sp. crude and undigested protein substrates were determined using the Kjeldahl method according to the AOAC method number 984.13 [19] with equipment of BÛCHI Digestion Unit K-424 (Switzerland). Crude proteins were estimated by multiplying the total nitrogen content by the factor of 6.25.

Lipids content was determined gravimetrically after the Soxhlet extraction of dried samples with hexane for 2 hours using Nahita Model 655 (Navarra, Spain).

As regards sugars, they were estimated by phenol-sulfuric acid method [20] using glucose as a standard.

### 2.4. Determination of Pigments Content: Chlorophylls and Carotenoids

Pigments were determined according to the method described by Lichtenthaler [21]. Two milliliters of culture was centrifuged at 5500 ×g for 5 min, and the supernatant was discarded and the pellet was mixed with 99.9% methanol and incubated in the dark for 24 hours at 45°C. After incubation, pigments content was determined measuring absorbances at 470, 652.4, and 665.2 nm, which were corrected for turbidity by subtracting absorbance at 750 nm.

### 2.5. Determination of Mineral Content

The analysis of sodium, potassium, calcium, magnesium, iron, copper, and zinc contents in* Amphora* sp. was carried out using the inductively coupled plasma optical emission spectrophotometer (ICP-OES) Model 4300 DV, PerkinElmer (Shelton, CT, USA), according to the method of AOAC 1999 [22]. Measurements were performed in triplicate and the results were the average of three values.

### 2.6. Determination of Fatty Acids Profile by GC-MS

Fatty acids methyl esters (FAME) were prepared by basic transesterification protocol. The extracted lipids were collected in 10 mL heptane and introduced into a 50 mL flask. A volume of 10 mL of KOH solution (11 g L^−1^) in methanol was added with a few antibumping beads. The mixture was boiled under reflux condenser for 10 min. Then, 5 mL of boron trifluoride (BF_3_), methanol complex (150 g L^−1^) was introduced through the condenser by a graduated syringe, and the mixture was boiled for 2 min. The mixture was cooled under room temperature and 15 to 20 mL of saturated sodium sulfate solution was added and shaken well. Furthermore, this solution was added until the liquid level reached the neck of the flask. After the phase separation, the upper layer (n-heptane) was collected by a Pasteur pipette and evaporated under nitrogen flow. The FAME were redissolved in 500 *µ*L hexane for GC-MS analysis.

The analysis of FAME was performed with a 6890 GC/5973 MSD GC/MS system from Agilent Technologies, equipped with an HP-INNO Wax Polyethylene Glycol capillary column (30 m length; 250 *µ*m diameter; 0.25 *µ*m film thickness). Helium was used as a gas carrier with a flow rate through the column of 1 mL/min. Column temperature was held initially for 1 min at 200°C and increased to 250°C at 10°C min^−1^ and was then held at 250°C for 15 min. The injector was kept at 260°C using a splitless mode, and then a sample of 1 *µ*L was injected. The ion source temperature was set at 230°C. The mass spectra were obtained in full scan mode MS, and scan range was (*m*/*z*): 50–600 atomic mass units (AMU) under electron impact (EI) ionization (70 eV). Data were exploited using the National Institute of Standards and Technology (NIST) Mass Spectral Search Program (version 2.0g).

### 2.7. Determination of the Total Phenolic Content

The total phenols content in microalga was determined by the Folin–Ciocalteu method [23]. Briefly, 0.2 mL of extract was mixed with 1 mL of Folin–Ciocalteu reagent (diluted 1 : 10, v/v) followed by the addition of 0.8 mL of sodium carbonate (7.5%, w/v). After incubation in the dark, the absorbance was measured at 760 nm. The total phenolic content of algal extract was expressed as mg of gallic acid equivalent per g of dry extract (mg GAE g^−1^ extract) using a calibration curve with gallic acid. All samples were analyzed in triplicate.

### 2.8. Determination of the Total Flavonoid Content

The total flavonoid content was determined according to the modified method of Zhishen et al. [24]. Briefly, 0.4 mL of the extract was mixed with 120 *µ*L of 5% sodium nitrite and 120 *µ*L of 10% aluminum chloride followed by the addition of 0.8 mL of 1 M sodium hydroxide. After the incubation of reaction mixture at room temperature for 6 min, absorbance was measured at 510 nm. The total flavonoids content in the extract was expressed in terms of catechin equivalent (mg g^−1^ of dry extract). All samples were analyzed in triplicate.

### 2.9. Extraction of Antioxidants

The extracts from the biomass samples were obtained by two solid-liquid extraction procedures inspired from the method of Goiris et al. [25]. In a first step, both apolar and polar compounds were extracted by 80% ethanol. For this, 2 g of freeze-dried biomass was ground using a pestle and mortar and extracted under agitation in the dark with 20 mL ethanol/water (4/1 v/v) mixture at 50°C for 1 hour. After centrifugation (4500 ×g, 10 min), the pellet was resuspended in 2 mL of the ethanol/water mixture and extracted for a second time with maceration. The two extracts were pooled and stored at −20°C prior to analysis. The second procedure aimed to separate polar from apolar compounds after sequential extraction in solvents with increasing polarity: hexane, ethyl acetate, and water. Freeze-dried biomass (2 g) was ground in a mortar and extracted with 20 mL of hexane for 1 hour at 50°C. After centrifugation, the pellet was resuspended in hexane and extracted for a second time, and both extracts were combined. The biomass pellet was subsequently extracted twice with 2 × 20 mL ethyl acetate using the same procedure and finally with 2 × 20 mL of water at 50°C for 1 hour. All extracts were concentrated under reduced pressure by a rotary evaporator to a dry condensed residue. The dried samples were weighed and then stored at −20°C prior to analysis.

### 2.10. Antioxidant Property

Four* in vitro* assays were used to evaluate the antioxidant property of the 80% ethanolic extract of* Amphora* sp.

#### 2.10.1. 2,2-Diphenylpicrylhydrazyl (DPPH) Free Radical Scavenging Assay

Free radicals scavenging activity was assessed according to Blois [26] with some modifications: 1 mL of the extract at different concentrations (0.06–1 mg mL^−1^) was mixed with 1 mL of 0.1 mM DPPH in ethanol and 450 *μ*L of 50 mM Tris-HCl buffer (pH 7.4). The solution was incubated at 37°C for 30 min, and the reduction of DPPH free radicals was evaluated by reading the absorbance at 517 nm. The DPPH scavenging activity is given as percentage and calculated according to the following equation:

(1) $$\begin{array}{c} \%\;\;DPPH\;\;scavenging=\left[\;\frac{\left(A_{control}-A_{test}\right)}{A_{control}}\;\right]\times 100, \end{array}$$

where *A*_control_ is the absorbance of the reaction medium and *A*_test_ is the absorbance of the reaction test containing the extract. The antioxidant activity of each extract was expressed as IC_50_, defined as the concentration of extract required to cause a 50% decrease in initial DPPH concentration. Butylated hydroxytoluene (BHT) was used as positive control, and each sample was analyzed three times.

#### 2.10.2. ABTS Radical Scavenging Activity

The Trolox equivalent antioxidant capacity (TEAC) assay, measuring the reduction of the 2,2-azinobis-(3-ethylbenzothiazoline-6-sulfonic acid) ABTS radical cation by antioxidants, was derived from the method of Katalinic et al. [27] with minor modifications. Briefly, ABTS radical cation (ABTS·+) was produced by reacting ABTS stock solution with 2.45 mM potassium persulfate, allowing the mixture to stand in the dark at room temperature for 12–16 hours before use. ABTS·+ solution was diluted with saline phosphate buffer (PBS, pH 7.4) to absorbance of 0.70 (±0.02) at 734 nm. After the addition of 2 mL of diluted ABTS·+ solution to 100 *μ*L of each extract, or Trolox standard, the reaction mixture was incubated for 30 min in a glass spectrophotometer cell at 30°C. The decrease in absorbance was recorded at 734 nm. Measurements were performed in triplicate. Radical scavenging activity was calculated using the formula below:

(2) $$\begin{array}{c} \%\;\;Inhibition=\left[\;\frac{\left(A_{control}-A_{test}\right)}{A_{control}}\;\right]\times 100, \end{array}$$

where *A*_control_ is the absorbance of the reaction medium and *A*_test_ is the absorbance of the reaction medium with the extract. Each sample was analyzed three times.

#### 2.10.3. Ferric Reducing Antioxidant Power (FRAP)

The reducing power of the extract was determined according to the method described by Yildirim et al. [28]. Briefly, 0.5 mL of 80% ethanolic extract solution at various concentrations (0.1–0.5 mg mL^−1^) was mixed with 1.25 mL of 0.2 M phosphate buffer (pH 6.6) and 1.25 mL of 1% (w/v) potassium ferricyanide K_3_[Fe(CN)_6_]. The mixture was incubated at 50°C for 20 min, and then 1.25 mL of 10% (w/v) trichloroacetic acid was added to the mixture which was then centrifuged at 3500 ×g for 10 min. The supernatant (1.25 mL) was mixed with 1.25 mL distilled water and 0.25 mL of 0.1% FeCl_3_ (w/v). The absorbance was measured at 700 nm. Ascorbic acid was used as a reference. The increase of the absorbance in the reaction indicates the increase of the iron reduction. The values presented are the mean of triplicate analysis.

#### 2.10.4. *β*-Carotene-Linoleic Acid Assay

The ability of the extract to prevent the bleaching of *β*-carotene was assessed as described by Koleva et al. [29]. A stock solution of *β*-carotene/linoleic acid mixture was prepared by dissolving 0.5 mg of *β*-carotene in 1 mL of chloroform, 25 *µ*L of linoleic acid, and 200 *µ*L of Tween 80. The chloroform was completely evaporated under vacuum in a rotary evaporator at 40°C, and then 100 mL of distilled water was added and the resulting mixture was vigorously stirred. The emulsion obtained was freshly prepared before each experiment. Aliquots (2.5 mL) of the *β*-carotene/linoleic acid emulsion were transferred to test tubes containing 0.1 mL of extract at different concentrations (0.1–0.5 mg mL^−1^). Following incubation for 2 h at 50°C, the absorbance of each test was measured at 470 nm. Vitamin C was used as a positive standard. The control tube contained no sample. Antioxidant activity in *β*-carotene bleaching model expressed as percentage was calculated with the following equation:

(3) β-carotene  bleaching  inhibition%=1−A0−AtA′0−A′t×100,

where *A*_0_ and *A*′_0_ are the absorbance of the sample and the control, respectively, measured at time zero and *A*_*t*_ and *A*′_*t*_ are the absorbance of the sample and the control, respectively, measured after 2 hours. Tests were carried out in triplicate.

### 2.11. Antibacterial Assays

The operative pathogens were Gram-positive (*Bacillus cereus* (ATCC11778),* Bacillus subtilis* (JN934392),* Micrococcus luteus* (ATCC4698), and* Staphylococcus aureus* (ATCC6538)) and Gram-negative (*Klebsiella pneumoniae* (ATCC13883),* Salmonella enterica* (ATCC43972)) bacteria. The antimicrobial activity of 80% ethanolic extract was evaluated using agar well diffusion method. The wells were then filled with 60 *µ*L of the extract at 20 mg/mL in 5% DMSO and 5% DMSO was used as negative control. Besides, plates were incubated for 24 hours at 37 ± 1°C for bacterial strains. Then, the diameters of inhibition zones (IZD) were measured.

### 2.12. Statistical Analysis

The data for biological and biochemical parameters are expressed as mean ± SD. Tests were carried out in triplicate. The IC_50_ values were calculated by Probit Analysis with a reliability interval of 95%.

## 3. Results and Discussion

### 3.1. Physicochemical Characterization of* Amphora* sp

Some physicochemical characteristics of* Amphora* sp. are presented in Table 1. The obtained results have shown that the biomass of* Amphora* sp. contains moderate amounts of lipids, proteins, carbohydrates, ashes, and minerals and an important percentage of chlorophyll a and carotenoids. The dry matter content of 7% is close to that found for other strains: 8% for* Amphora* sp. CTM 20023 [30] and 5.9% for* Amphora coffeaformis* [31]. However, the lipids content of* Amphora* sp. of 11.14% DW was relatively lower than the values published for other strains of* Amphora* [32], and it was higher than that of* Amphora coffeaformis* (6.9% DW) [31]. A study on eight marine species of diatoms revealed various lipid contents ranging from 2.4 to 21.3% [33].

The proteins level for* Amphora* sp. (27.62%) was lower than that found for other strains of* Amphora*; 54% for CTM 20023 [30] and 30–40% for* Amphora* sp. [32]. Indeed, the proteins level was in agreement with the published values range of 12–42% for some microalgae [34] and higher than the contents found for* Amphora coffeaformis* [31].

The total sugars content of* Amphora* sp. was 12.60% DW, which is consistent with that of some microalgae (5–23% DW) [34]. Nevertheless,* Amphora coffeaformis* and some diatoms contain a high amount of sugars in the range of 13.5–16.4% [31].

With respect to the ash content of* Amphora* sp. (37.78% DW), it is in line with that found for another* Amphora* strain (30% DW) cultivated at salinity ranging between 15 and 35 p.s.u. [32]. Ash content exceeds 50% (55.8 to 67.9%) of the dry weight for some diatoms [31].* Amphora* sp. has moderate amounts of sodium, potassium, calcium, and magnesium.

Moreover,* Amphora* sp. was found to be rich in chlorophyll, mainly chlorophyll a, and carotenoids. In fact, its chlorophyll content reached almost 5% of the dry matter, which is in agreement with other research works [30]. Furthermore, the carotenoids content was prominent (1.083% DW). Bacillariophyceae exhibited high levels of carotenoids, namely, *β*-carotene and xanthophylls, thus indicating a powerful antioxidant activity [35].

### 3.2. Fatty Acids Composition of* Amphora* sp

In salinity of 100 p.s.u., on F/2 medium, the fatty acids profile of* Amphora* sp. was composed of saturated, monounsaturated, and polyunsaturated fatty acids (SFA, MUFA, and PUFA), respectively (Table 2). The C16 fatty acids series (C16:0, C16:1, C16:2, and C16:3) represented more than 75%. Moreover, palmitic acid (C16:0) and palmitoleic acid (C16:1) accounted together for more than 72.51% of fatty acids. SFA were present at a high level, 41.31%, with 27.41% of palmitic acid. Hence,* Amphora* sp. could be a suitable producer of SFA, which are easily convertible to biodiesel [36].* Amphora* sp. is rich in MUFA with the dominance of palmitoleic acid (45.09%) and contains an important percentage of PUFA (6.03%). High levels of palmitoleic acid and other bioactive fatty acids were also detected in the fusiform morphotype of the Bacillariophyceae* Phaeodactylum tricornutum* [37]. Eicosapentaenoic acid C20:5 (EPA) was produced in a noticeable level (2.367%). Indeed, it is known that EPA is an important PUFA for health protection from many pathologies, including cardiovascular diseases [38] and cancer [39].

### 3.3. Phytochemical Composition

The total phenols and flavonoids contents of* Amphora* sp. extracts are summarized in Table 3. The 80% ethanolic extract of this Bacillariophyceae showed the highest phenols and flavonoids contents, 38.27 ± 2.21 mg GAE g^−1^ extract and 17.69 ± 0.70 mg CE g^−1^ extract, respectively, compared to the extracts obtained by ethyl acetate, hexane, and water. The phenolic content of ethanol extracts of some Moroccan marine microalgae was found to range from 8.2 to 32 mg GAE g^−1^ extract [40]. The authors noted that the highest phenolic content was found in the extract of* Nannochloropsis gaditana* with 32 mg GAE g^−1^ extract. The extracts of* Dunaliella* sp.,* Phaeodactylum tricornutum*, and* Navicula* sp. also contained high phenolic content of more than 15 mg GAE g^−1^ extract [40].

The flavonoid content in the 80% ethanolic extract of* Amphora* sp. was 3.22 mg CE g^−1^ DW, which is sixfold higher than that found for* Amphora* CTM 20023 [30].

The high levels of polyphenols and flavonoids in the* Amphora* sp. 80% ethanolic extract may be due to the culture conditions under the high salinity of 100 p.s.u. and the extraction conditions.

### 3.4. Antioxidant Property of* Amphora* sp

**Figure 1:**
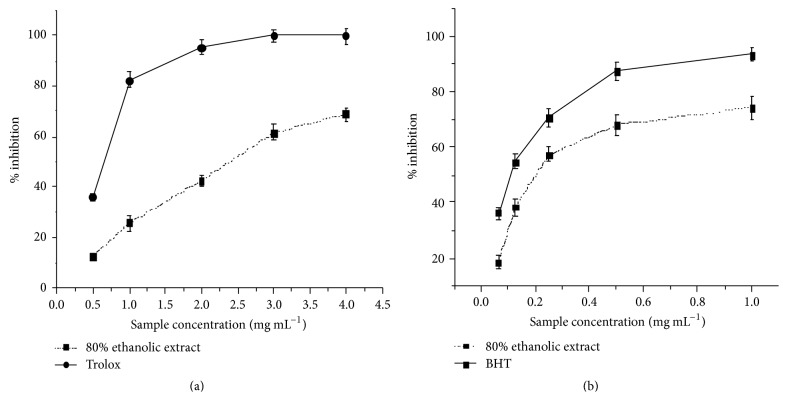
Anti-free radical effects of* Amphora* sp. 80% ethanolic extract for ABTS (a) and DPPH (b) compared to respective standards (Trolox and BHT).

Due to its highest content of phenols and flavonoids, the 80% ethanolic extract was used to check antioxidant activity through four* in vitro* assays: the DPPH and ABTS radical scavenging capacities, the ferric reducing antioxidant power, and the *β*-carotene bleaching inhibition tests, using different concentrations of the extract. The inhibition percentages of the radicals DPPH and ABTS by the extract and standards (Trolox and BHT) were found to be concentration-dependent (0.065 to 1 mg mL^−1^ for DPPH test and 0.5 to 4 mg mL^−1^ for ABTS test) as shown in Figure 1. The deduced values of IC_50_ are presented in Table 4. A lower value of IC_50_ indicates a high antioxidant activity.* Amphora* sp. 80% ethanolic extract exhibited high antiradical power with an IC_50_ value of 0.23 ± 0.07 mg mL^−1^ for DPPH scavenging activity and an IC_50_ value of 2.61 ± 0.64 mg mL^−1^ for ABTS radical scavenging capacity. Some authors have found a strong correlation between the phenolic contents and the antioxidant activity [41].

**Figure 2:**
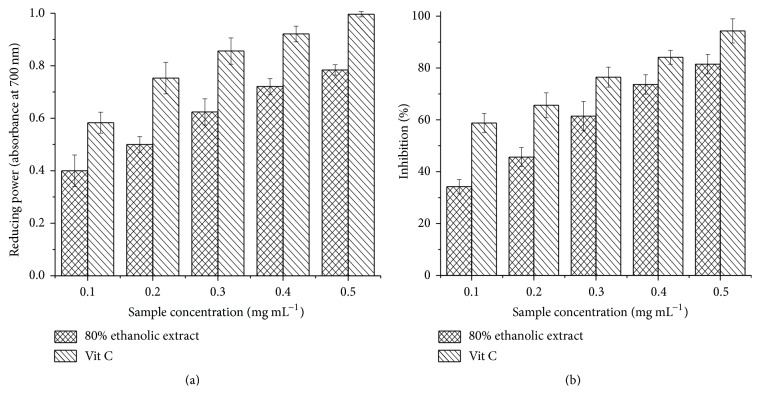
Ferric reducing antioxidant power (FRAP) (a) and beta-carotene bleaching test (b) for 80% ethanolic extract of* Amphora* sp. compared to vitamin C (Vit C) as a standard.

The FRAP assay is based on the ability of phenols to reduce yellow ferric tripyridyl triazine complex (Fe(III)-TPTZ) to blue ferrous complex (Fe(II)-TPTZ) by the action of electron-donating antioxidants [42].  Figure 2(a) shows a significant reducing power compared to the vitamin C used as a reference. The reducing power was also dependent on the extract concentration.

The effect of antioxidants on DPPH free radical scavenging was considered to be emanating from their hydrogen-donating ability. In this investigation, the 80% ethanol extract was proven to exhibit a strong effect of DPPH free radical scavenging. In fact, it reached 74.34% of inhibition at 1 mg of extract, which is considered higher than the methanol extract of some Bacillariophyceae tested at 2 mg of the extract (22.7%, 31.6%, and 76.6% for* Amphora coffeaeformis*,* Navicula* sp., and* Achnanthes longipes*, resp.) [31]. The IC_50_ value for ABTS scavenging capacity of* Amphora* sp. extract was 2.6 mg mL^−1^. Many research studies [43] have mentioned IC_50_ ABTS scavenging capacities for two brown algae of 5.29 mg mL^−1^ for* Sargassum binderi* and 15.2 mg mL^−1^ for* Turbinaria conoides*.

The *β*-carotene bleaching inhibition and the reducing power tests (absorbance at 700 nm) were measured at concentrations of extract between 0.1 and 0.5 mg mL^−1^, showing its dependency on concentration (Figure 2(b)). The IC_50_ value of *β*-carotene bleaching test (Table 4) was strong (0.21 ± 0.05 mg mL^−1^), with about twice the IC_50_ value of vitamin C used as the standard. The absorbance at 700 nm reached a value of 0.78 at a concentration of 0.5 mg mL^−1^ of the extract, which is considered high, indicating a good antioxidant activity. Therefore,* Amphora* sp. extract was confirmed to have a strong antioxidant capacity. The multiple regression analysis has proven that both carotenoid and phenolic contents significantly contribute to the explanation of the variation in FRAP and TEAC activity of the extracts. The regression coefficients indicate that phenols and carotenoids are of similar importance in explaining variation in the antioxidant activity [25].

### 3.5. Antibacterial Assay

In the present study, the antibacterial activity of* Amphora* sp. 80% ethanolic extract was screened against six bacteria and their potency was qualitatively and quantitatively analyzed by the diameters of the inhibition zones (IZD) as summarized in Table 5. The 80% ethanolic extract of* Amphora* sp., at a concentration of 20 mg mL^−1^, was active against Gram-positive bacteria,* Micrococcus luteus* and* Staphylococcus aureus*, and inactive against both strains of* Bacillus* (*cereus* and* subtilis*). This extract was active against Gram-negative bacteria tested:* Klebsiella pneumonia* and* Salmonella enterica*. The extract exhibited strong activity against* Micrococcus luteus*,* Staphylococcus aureus*, and* Klebsiella pneumoniae*. The IZD for* Klebsiella pneumoniae* and* Staphylococcus aureus* of 16 mm and 20 mm, respectively, were stronger than those found for the ethanolic extract of a strain of* Amphora* sp. cultured in standard Walne's medium (10 mm and 10.5 mm), respectively [44]. This difference may be due to the culture conditions. According to some authors, the antimicrobial activity of algae depends on the species and the extraction efficiency of the active compounds as well as survival conditions [45].

The extract used in this study was found to be rich in a variety of compounds: lipids and fatty acids, carbohydrates, polysaccharides, polyphenols, flavonoids, pigments, and carotenoids, which can be indicative for having antibacterial activity as mentioned for several microalgae [46]. The antimicrobial activity of microalgae has been attributed to compounds belonging to several chemical classes including indoles, terpenes, acetogenins, phenols, fatty acids, and volatile halogenated hydrocarbons [47, 48]. The mechanisms of action of some antimicrobial agents were described; carotenoids digest the cell wall [49]; flavonoids increase permeability of the inner bacteria [50]; polyphenols participate in enzyme inhibition, substrate deprivation, complexing with cell wall, and membrane disruption [8]; polysaccharides inhibit hyaluronidase [8], while fatty acids and lipids cause the disruption of the cellular membrane [51]. The antimicrobial activity of supercritical extracts obtained from the microalga* Chaetoceros muelleri* was related to its lipids composition [52]. Fatty acids were reported to be bioactive compounds having antibacterial activity in some diatoms* Phaeodactylum tricornutum* [51, 53] and* Skeletonema costatum* [54] and other microalgae* Haematococcus pluvialis* [55] and* Dunaliella salina* [56].

Microalgae extracts should be tested as additives for food and feed formulations, in an attempt to replace the currently used antimicrobial compounds of synthetic origins, including subtherapeutical doses of antibiotics employed as a prophylactic measure in animal breeding [9].

## 4. Conclusion

*Amphora* sp., a brown microalga cultivated in high salinity medium of 100 p.s.u., was confirmed to contain a moderate percentage of proteins, lipids, sugars, and minerals and important levels of polyphenols, flavonoids, chlorophyll, carotenoids, and bioactive fatty acids as EPA. Besides, this microalga has important antioxidant and antibacterial activities. Prominent contents of bioactive compounds in this microalga are in favor of its possible valorization in food additives and pharmaceutical or cosmetic products. Moreover, its richness in saturated fatty acids allows its eventual use for biodiesel production.

## Figures and Tables

**Table 1:** Physicochemical characteristics of *Amphora* sp.

Component	*Amphora* sp.
Dry matter (% FW)	7 ± 0.45
Proteins (% DW)	27.62 ± 0.3
Lipids (% DW)	11.14 ± 0.19
Total sugars (% DW)	12.60 ± 0.76
Ashes (% DW)	37.78 ± 0.43
Chlorophyll a (% DW)	4.945 ± 0.2
Chlorophyll b (% DW)	0.666 ± 0.05
Carotenoids (% DW)	1.083 ± 0.05
Sodium (g Kg^−1^ DW)	1.125 ± 0.2
Potassium (g Kg^−1^ DW)	0.485 ± 0.05
Calcium (g Kg^−1^ DW)	0.584 ± 0.05
Magnesium (g Kg^−1^ DW)	0.747 ± 0.1
Iron (g Kg^−1^ DW)	0.016 ± 0.002
Copper (g Kg^−1^ DW)	0.008 ± 0.001
Zinc (g Kg^−1^ DW)	0.008 ± 0.001

Data are expressed as mean ± standard deviation of triplicates. FW: fresh weight; DW: dry weight.

**Table 2:** Fatty acids composition of *Amphora* sp. (% of total fatty acids).

Fatty acids	*Amphora* sp.
C14:0	3.623 ± 0.3
C15:0	3.418 ± 0.3
C16:0	27.427 ± 0.5
C17:0	1.664 ± 0.4
C18:0	1.974 ± 0.3
C20:0	0.734 ± 0.2
C24:0	2.468 ± 0.2

∑SFA	**41.308 ± 0.8**

C14:1	3.386 ± 0.3
C16:1	45.089 ± 0.8
C17:1	0.521 ± 0.1
C18:1	3.658 ± 0.3

∑MUFA	**52.654 ± 1.2**

C16:2	1.603 ± 0.2
C16:3	0.924 ± 0.3
C18:2	0.432 ± 0.1
C20:4	0.712 ± 0.2
C20:5	2.367 ± 0.3

∑PUFA	**6.038 ± 0.5**

∑SFA: total saturated fatty acids; ∑MUFA: total monounsaturated fatty acids; ∑PUFA: total polyunsaturated fatty acids.

**Table 3:** Extraction yields and polyphenols and flavonoids contents in extracts from *Amphora* sp. with different solvents.

Solvent	Yield (% DW)	Polyphenols (mg GAE g^−1^ extract)	Flavonoids (mg CE g^−1^ extract)
80% ethanol	18.20 ± 0.21	38.27 ± 2.21	17.69 ± 0.70
Hexane	14.95 ± 0.25	11.47 ± 0.60	4.76 ± 0.15
Ethyl acetate	2.78 ± 0.12	14.04 ± 0.31	5.25 ± 0.30
Water	23.61 ± 0.30	13.34 ± 0.24	4.27 ± 0.20

Data are expressed as mean ± standard deviation of triplicates. GAE: gallic acid equivalent; CE: catechin equivalent.

**Table 4:** IC_50_ values for antioxidant capacities of *Amphora* sp. extract compared to standards.

Activity	Extract (mg mL^−1^)	Standard
DPPH radical scavenging activity	0.23 ± 0.07	0.11 ± 0.03
ABTS radical scavenging activity	2.61 ± 0.64	0.54 ± 0.08
*β*-Carotene bleaching assay	0.21 ± 0.05	0.09 ± 0.01

Data are expressed as mean ± standard deviation of triplicates.

**Table 5:** Antibacterial activity of *Amphora* sp. 80% ethanolic extract.

Strains	Gram +/−	IZD (mm)
		80% ethanol extract of *Amphora* sp.
*Bacillus cereus* (ATCC11778)	+	0 ± 0
*Bacillus subtilis* (JN934392)	+	0 ± 0
*Micrococcus luteus* (ATCC4698)	+	16 ± 0.5
*Staphylococcus aureus* (ATCC6538)	+	20 ± 2
*Klebsiella pneumoniae* (ATCC13883)	−	16 ± 0.5
*Salmonella enterica* (ATCC43972)	−	12 ± 1

The data are expressed as mean ± SD (*n* = 3). IZD: inhibition zone diameter.
